# Pricing, price revision, and clinical value of anticancer drugs in Japan: a retrospective observational study

**DOI:** 10.3389/fphar.2025.1641775

**Published:** 2025-11-18

**Authors:** Jiaqi He, Haibo Wei, Yue Yang

**Affiliations:** 1 School of Business Administration, Shenyang Pharmaceutical University, Shenyang, China; 2 School of Pharmaceutical Sciences, Tsinghua University, Beijing, China; 3 Key Laboratory of Innovative Drug Research and Evaluation, National Medical Products Administration, Beijing, China; 4 School of Biomedical Engineering, Hainan University, Sanya, China

**Keywords:** anticancer drugs, pricing, price revision, clinical value, Japan

## Abstract

**Introduction:**

The high cost of anticancer drugs has raised concerns due to the financial burden on patients and pressure on healthcare systems. We examine the relation between pricing premiums, price revision, and clinical value for anticancer drugs approved in Japan.

**Methods:**

We included anticancer drugs approved in Japan from 2013 to 2023. Differences in clinical value, as measured by the European Society for Medical Oncology Magnitude of Clinical Benefit Scale (ESMO-MCBS), among drugs receiving different premium proportions, were analyzed using the Mann-Whitney U test. Pearson correlation coefficients were calculated between treatment costs and clinical outcomes and value, including progression-free survival (PFS) gain, overall survival (OS) gain, objective response rate (ORR) gain and ESMO-MCBS. We also compared the magnitude of price revisions and price reductions with different clinical values. We used regression analysis to explore the association between clinical value, price revision, and influencing factors.

**Results:**

Our cohort included 94 anticancer drugs and 119 key clinical trials. 39 drugs (41.49%) received premium pricing, while 55 drugs (58.51%) did not. Drugs with pricing premiums had higher ESMO scores than those without (3 vs. 2; P = 0.0013), with ESMO scores showing an increasing trend as the proportion of additional scores rose. Pearson correlation analysis revealed no significant association between daily treatment cost and ESMO score (r = 0.025, P = 0.79), PFS gain (r = −0.12, P = 0.37), OS gain (r = −0.25, P = 0.066), or ORR gain (r = 0.049, P = 0.8). Additionally, no significant differences were observed between high clinical value drugs (ESMO = 4–5) and low-to-medium clinical value drugs (ESMO = 1–3) in median price revision percentage and median price reduction percentage (−0.02% vs. 0.00%, P = 0.65; −9.86% vs. −13.55%, P = 0.607). Moreover, multivariate linear regression analysis revealed no association between ESMO scores, premium proportion, PFS gain, OS gain, ORR gain, approval year (P = 0.32; P = 0.06; P = 0.71; P = 0.06; P = 0.50; P = 0.51).

**Discussion:**

Our study found that Japan’s price premium reflects clinical value and provides incentives for high-clinical-value anticancer drugs. However, during both initial pricing and subsequent revision phases, there is room for improvement in aligning drug prices with clinical value. Policymakers should further refine pricing and revision systems to more effectively promote clinical value-driven drug pricing.

## 1 Introduction

The global cancer burden has continued to rise, becoming a major public health issue. The 2022 Global Cancer Statistics Report shows that in 2022, there were nearly 20 million new cancer cases worldwide, with 9.7 million deaths (Bray et al., 2024). Meanwhile, spending on cancer drugs is also growing rapidly. Global spending on cancer medicine increased to $223 billion in 2023, $25 billion more than in 2022, and is projected to reach $409 billion by 2028 (IQVIA, 2024). This trend has not only accelerated the rapid development of new technologies in cancer drugs but has also drawn increasing attention to the high cost of these medications.

To improve patients’ access to cancer drugs and alleviate the financial burden on healthcare systems, health policymakers worldwide have implemented various measures, with the establishment of reasonable pricing systems being regarded as an effective strategy. Value-based pricing has gradually become a global trend in pharmaceutical pricing policies (WHO, 2019). Previous studies have explored the correlation between the prices of anticancer drugs and their clinical efficacy, suggesting that, in principle, drug prices should reflect their clinical value (Robinson et al., 2018; Broder and Ortendahl, 2018; Augustovski and McClellan, 2019; Howard et al., 2015). In Japan, drug prices are unrelated to treatment outcomes measured by OS, and the correlation between the initial pricing of cancer drugs and their clinical value is lower than in China and South Korea (Satoh et al., 2018; Pan et al., 2024).

Japan has established a unique drug pricing system to promote innovation and ensure patients can access high-clinical-value drugs. During the pricing process, a premium system is applied to add a certain percentage to the base price of drugs, incentivizing pharmaceutical companies to develop products with high clinical value (MHLW, 2025a). Therefore, the extent to which the premium system can accurately identify medicines of high clinical value will, to a certain degree, influence whether the final pricing is reasonable and will also affect the enthusiasm of enterprises for research and development investment.

Additionally, Japan periodically adjusts drug prices based on actual market transaction prices. Although this mechanism helps control healthcare expenditure, uncertainty remains as to whether it can provide relative protection for drugs of high clinical value during the revision process. Japan officially introduced a cost-effectiveness assessment system in 2019 to further enhance the scientific rigor of drug pricing. However, its application is currently limited to the post-market price revision phase and has not been incorporated into the initial pricing stage (Shiroiwa, 2020). This approach differs significantly from the United Kingdom’s introduction of health technology assessment during the initial market launch phase and Germany’s additional benefit assessment (Cherla et al., 2020; Gandjour, 2025).

Previous studies have examined the correlation between cancer drug prices and their clinical value in Japan. But research on the rationality of the premium system in drug pricing and whether the drug price revision system possesses value protection characteristics remains lacking. Therefore, in this study, we assess the relation between premium pricing, price revision systems, and clinical value in Japan.

## 2 Methods

### 2.1 Sample identification

Given that Japan implements a biennial drug price revision system in principle, this study involves both initial pricing and subsequent price revision. We obtained information on newly approved cancer drugs from the Pharmaceuticals and Medical Devices Agency (PMDA) between January 2013 and March 2023. We reviewed the latest labels published by the Ministry of Health, Labour, and Welfare (MHLW, 2025b) and selected indications approved for the first time during this period.

During the drug selection process, two exclusion criteria were applied to ensure data completeness and comparability. Firstly, drugs without information on their initial market launch could not provide accurate data on initial prices or subsequent revision, making quantitative analysis infeasible. Secondly, drugs intended for local irradiation of lesions differ in clinical use from systemic therapies and may bias the results. Consequently, two drugs without initial launch information and two drugs used for local irradiation were excluded.

### 2.2 Clinical outcomes and value extraction

To assess the clinical value of the drugs, we extracted the following data. We first used the ESMO-MCBS to evaluate the clinical value of each indication. Solid tumors and hematological malignancies were assessed separately, with reference to the official ESMO website and the latest criteria published by the ESMO Working Group. The ESMO website is updated periodically; therefore, when the most recent ESMO-MCBS scores for certain indications were not publicly available, we estimated them based on the latest clinical trial evidence using the official ESMO-MCBS evaluation forms and online tutorials.

For therapies supported by randomized controlled trials (RCTs), we extracted the median overall survival (OS) and progression-free survival (PFS) for both the treatment and control groups. The percentage increase in OS and PFS, derived from the difference between these medians, was used as an indicator of clinical benefit. For single-arm trials, we extracted the objective response rate (ORR) reported for the treatment group.

All clinical endpoints (PFS gain, OS gain, ORR gain) were then extracted and analyzed as absolute differences between the experimental and control arms, consistent with the ESMO-MCBS methodology, which quantifies clinical benefit based on absolute improvements.

### 2.3 Daily costs calculation

We extracted relevant data and information, including initial drug prices, premium proportions, patient population, and indications, from the New Drug Pricing Table (New Drug List) published by the Ministry of Health, Labour, and Welfare (MHLW, 2025b). Additionally, we obtained the latest drug prices (as of April 2025) from the National Health Insurance Drug Price List and extracted dosage and administration information from the drug labels. Due to variations in drug dosage, administration frequency, and treatment duration, using price alone as an assessment criterion may not accurately reflect the actual economic burden borne by patients during treatment. Consistent with previous studies (Zhang et al., 2022), we calculated the daily treatment cost based on the total dosage required for a complete treatment cycle (Vokinger et al., 2020). The maintenance dosage is selected when a drug has both an initial and a maintenance dosage within a complete treatment cycle. If a drug is available in multiple formulations, the formulation with the lowest price was prioritized for calculation. For medications requiring dosage based on body weight or body surface area, similar to previous studies, we assumed an average adult body weight of 70 kg and a body surface area of 1.7 m^2^ (Ratain, 1998; Prasad et al., 2017).

### 2.4 Statistical analysis

We used the Mann-Whitney U test to compare the relationship between different premium percentages and clinical value (ESMO-MCBS), as well as the differences in price revision and price reduction between drugs with different levels of clinical value (ESMO score: high value defined as 4-5, low-to-moderate value defined as 1–3). To assess the correlation between drug premium and clinical value, we calculated the Pearson correlation coefficients between the daily treatment cost of the drug and clinical outcomes (OS gain, PFS gain and ORR gain) and clinical values (ESMO-MCBS). Additionally, the study employed multivariate linear regression analyses to investigate the relationships between various indicators of clinical value and drug price revision magnitude.

Three robustness tests were performed to assess the reliability of the results. Firstly, we applied ordinary least squares (OLS) regression and OLS models with HC3 robust standard errors to examine the potential impact of type I error on the results. Secondly, we performed median quantile regression (QR) to evaluate the stability of estimates across different points of the distribution. Thirdly, the variance inflation factor (VIF) for each variable was calculated to assess multicollinearity issues in the model. All statistical analyses and figure generation were done in R Studio. P-values less than 0.05 were considered statistically significant.

## 3 Results

### 3.1 Characteristics of sample anticancer medicines and indications

**FIGURE 1:**
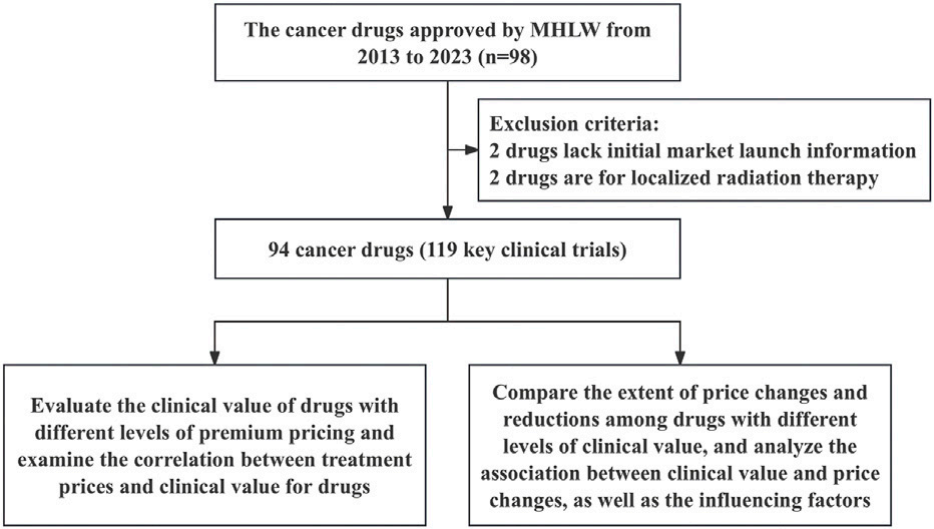
The framework of inclusion for anticancer drugs.

94 anticancer drugs and their associated 119 pivotal clinical trials were included in this study (Figure 1). Among all the sampled drugs, the most common indication was lymphoma (n = 17, 18.09%), followed by non-small cell lung cancer (n = 15, 15.96%). Regarding approvals, 46 (48.94%) and 48 (51.06%) of the drugs were approved for anticancer indications between 2013–2017 and 2018–2023, respectively. Most indications were approved through routine approval (n = 80, 85.11%). Regarding pivotal clinical trial design, 61.34% used randomized controlled trials, and 38.66% used single-arm trials. Most of the pivotal clinical trials for anticancer drugs were distributed in phase III clinical trials (n = 68, 57.14%) (Table 1).

**TABLE 1:** Characteristics of indications and pivotal trials in the study sample**.**

Characteristic	Number	Percent (%)
Drug	94	100
Cancer type		
Lymphoma	17	18.09
Non-small-cell lung cancer	15	15.96
Melanoma	8	8.51
Multiple Myeloma	8	8.51
Prostate cancer	7	7.45
Breast cancer	6	6.38
Others	33	35.11
Approval year		
2013–2017	46	48.94
2018–2023	48	51.06
Approval type		
Conditional approval	14	14.89
Regular approval	80	85.11
Total of pivotal trial	119	100
Study design		
Randomized	73	61.34
Single-arm	46	38.66
Clinical trial phase		
Phase 4	1	0.84
Phase 3	68	57.14
Phase 1 or 2	50	42.02

### 3.2 Difference between premium and clinical value

39 drugs (41.49%) received different degrees of premium, and 55 drugs (58.51%) did not receive up premium, corresponding to the number of pivotal clinical trials of 46 and 73 (38.66% vs. 61.34%), respectively.

**FIGURE 2:**
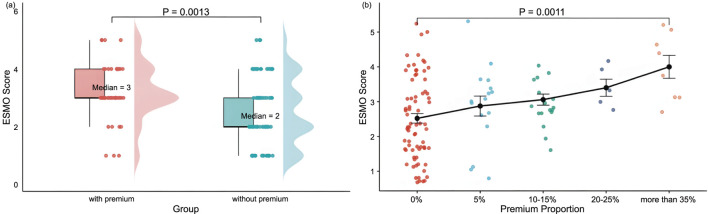
Distribution and comparison of ESMO Scores Between Groups: **(a)** With premiums (n = 46) and without premiums (n = 73), and **(b)** With varying price premium proportion-The distribution of price premium proportions was as follows:0% (n = 73), 5% (n = 16), 10%–15% (n = 17), 20%–25% (n = 5), and more than 35% (n = 8). Note: With premium, premium proportion more than 5%; without premium, premium proportion of 0%. Premiums include innovation premium, clinical utility premium, market size premium, pediatric drug premium, advanced therapy premium, and Sakigake Designation premium, in the context of Japan’s drug price premium system.

The median ESMO score was significantly higher in the premium group than in the non-premium group (3 vs. 2, P = 0.0013). Drugs were categorized into five groups based on the premium percentage: 0%, 5%, 10%–15%, 20%–25%, and ≧30%. The results showed that the mean ESMO scores for these groups were 2.52, 2.88, 3.06, 3.40, and 4.00, respectively. ESMO scores tended to increase significantly with the increase in the addition percentage, and a notable difference was observed between the non-premium group and the ≥30% premium group (median: 2 vs. 4; P = 0.0011) (Figure 2).

### 3.3 Correlation between daily cost and clinical outcome and value

**FIGURE 3:**
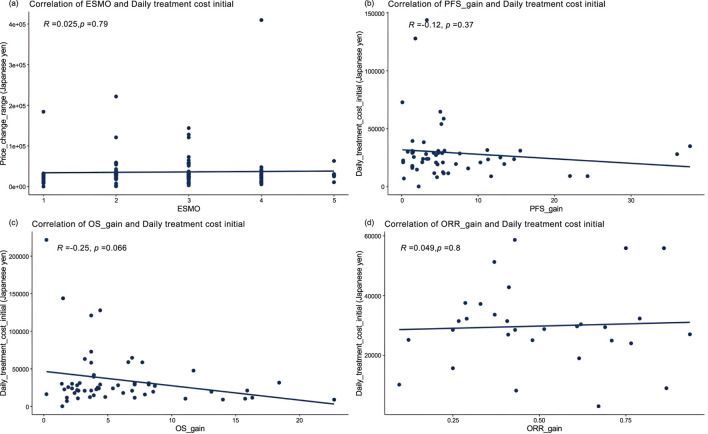
Correlation between cancer therapy daily costs and percentage improvement in **(a)** ESMO scores (n = 119), **(b)** progression-free survival (PFS) of randomized controlled trials (n = 55), **(c)** overall survival (OS) of randomized controlled trials (n = 57), and **(d)** objective response rate (ORR) of single-arm trials (n = 31).

Among 119 pivotal clinical trials, Pearson correlation analysis revealed no association between ESMO score and daily treatment cost (r = 0.025, P = 0.79) (Figure 3). Further stratification by add-on status showed no significant correlation between ESMO score and daily treatment cost in either the with or without premium groups (r = −0.0054, P = 0.96; r = −0.12, P = 0.42) (Supplementary Figure S2).

Among the 73 pivotal randomized controlled trials (RCTs), PFS data were available in 55 (75.34%), the PFS gain ranged from 0.1% to 37.7%, and OS data in 57 (78.08%), OS gain ranged from 0.2% to 22.7%. Pearson analysis showed no correlation between PFS gain, OS gain and daily treatment cost (r = −0.12, P = 0.37; r = −0.25, P = 0.066). Further analysis based on whether add-on therapy was administered, Pearson results indicated, with premium and without premium, respectively, no correlation PFS gain (r = −0.073, P = 0.67; r = −0.16, P = 0.5), and OS gain (r = −0.36, P = 0.082; r = −0.063, P = 0.73) all exhibited weak negative correlations with daily treatment costs.

For the 31 therapies supported solely by single-arm trials, ORR gain was uncorrelated with daily treatment cost (r = 0.049, P = 0.8). However, ORR gain in the with premium group showed a significant positive correlation with daily treatment cost (r = 0.59, P = 0.01), while the without premium group exhibited a significant negative correlation (r = −0.59, P = 0.034).

### 3.4 Correlation between the magnitude of drug price revision and clinical value

**FIGURE 4:**
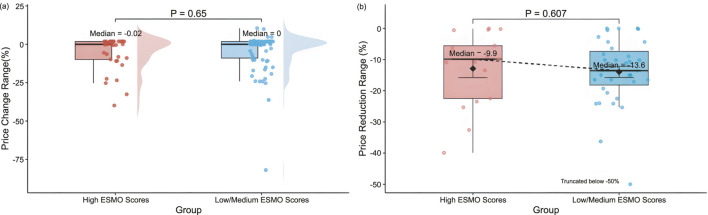
Distribution of **(a)** price revision magnitude by ESMO score Groups (High, 4-5 points (n = 33); Low/Medium, 1-3 points (n = 86)), and **(b)** price reduction range by ESMO score groups (High, 4-5 points (n = 17); Low/Medium, 1-3 points (n = 35)).

According to the ESMO scoring criteria, the 119 pivotal clinical trials included in this study were categorized into the low/medium clinical value group (1-3 points) and the high clinical value group (4-5 points). The two groups contained 86 (72.27%) and 33 (27.73%) clinical trials, respectively. There was no significant difference between the two groups regarding the magnitude of drug price revision (median: 0.00% vs. −0.02%, P = 0.65) (Figure 4). Further comparing the magnitude of price reductions between the two groups, the magnitude was slightly higher in the low/medium clinical value group than in the high clinical value group, but still not significantly different (median: −13.55% vs. −9.86%, P = 0.607) (Figure 4).

We used multivariate linear regression analysis to assess the relationship between ESMO scores of pivotal clinical trials of drugs and the price revision magnitude of drugs (Table 2). The results showed that ESMO scores, premium proportion, PFS gain, OS gain, ORR gain, approval year were not significantly associated with the extent of price revision (P = 0.32; P = 0.06; P = 0.71; P = 0.06; P = 0.50; P = 0.51).

**TABLE 2:** Regression analysis of the impact of ESMO scores on drug price revision magnitude.

Variables	Price revision magnitude						
	OLS		Robust OLS HC3		Quantile reg		VIF
	Coefficient (95%)	P Value	Coefficient (95%)	P Value	Coefficient (95%)	P Value	
ESMO	−2.97 (−8.99–3.04)	0.32	−2.97 (−9.30–3.35)	0.34	−5.44 (−14.75–3.86)	0.81	1.41
PFS gain	−0.19 (−1.18–0.81)	0.71	−0.19 (−0.74–0.37)	0.50	0.04 (−3.58–3.66)	0.24	1.25
OS gain	−0.94 (−1.91–0.02)	0.06	−0.94 (−2.62–0.74)	0.26	−0.08 (−5.66–5.50)	0.98	1.05
ORR gain	0.63 (−1.24–2.50)	0.50	0.63 (−11.04–12.30)	0.91	0.18 (−471.84–472.21)	0.97	1.11
Approval year	0.83 (−1.72–3.40)	0.51	0.84 (−2.47–4.14)	0.61	−0.64 (−6.17–4.90)	0.99	1.07
Premium proportion	39.29 (−1.34–79.93)	0.06	39.30 (19.49–59.10)	0.0003	34.52 (−39.97–109.00)	0.81	1.35

OLS, Ordinary Least Square; HC3, Ordinary Least Squares with HC3 robust standard errors; QR, median, Median Regression based on Quantile Regression; VIF, Variance Inflation Factor. The OLS, model was used as the primary analysis, while the Robust OLS HC3 was conducted as a robustness check. Quantile Regression (QR, median) was included as an additional sensitivity analysis. VIF, values were computed from the OLS, model (for each predictor regressed on the remaining predictors). VIF≈1 indicates no collinearity; 2-5 modest; >10 severe. Significance levels: *p < 0.05; **p < 0.01; ***p < 0.001.

To test the robustness of the main model, we constructed several sensitivity analyses. Firstly, we re-estimated the main regression model using ordinary least squares (OLS), incorporating heteroscedasticity-robust standard errors (HC3 correction) to mitigate potential Type I error risks. Secondly, median quantile regression was employed to assess the robustness of estimation outcomes across varying distribution positions. Findings demonstrated that conclusions under different model specifications remained consistent with the primary analysis: neither the ESMO score nor other controlled variables exhibited significant correlation with the magnitude of price revision (Supplementary Table S1), thereby further enhancing the credibility of the research findings. Additionally, in the regression model, the variables’ variance inflation factors (VIF) are all less than 1.5, indicating that the model does not have serious multicollinearity problems and that the model estimation results have good robustness.

## 4 Discussion

Our study initially examined the relationship between whether a drug received a premium, its proportion, and its clinical value. A total of 94 anticancer drugs and 119 pivotal clinical trials were included. The results showed that drugs awarded a premium had significantly higher ESMO scores than those without a premium, and ESMO scores increased significantly with greater premium proportions. These findings suggest that anticancer drugs granted a premium at the initial pricing stage generally have higher clinical value, and the magnitude of the premium can reflect this value to some extent. This is consistent with the original policy intention of Japan’s premium system.

In Japan, the premium system evaluates the clinical characteristics of new drugs using a quantitative scoring framework. This framework considers multiple dimensions, including clinical effectiveness, safety, and improvements in patients’ quality of life, to determine the appropriate premium proportion (MHLW, 2023). The premium determination process, the decision to grant a premium and the specific proportion are made collectively by the Drug Pricing Organization, composed of multidisciplinary experts, which helps ensure a degree of scientific rigor and fairness.

We further assessed the relationship between initial drug pricing and clinical value (PFS, OS, ORR, ESMO). Whether in the overall sample or in subgroup analyses by surcharge status, clinical value showed no correlation or only a weak correlation with daily treatment costs. This result is consistent with previous studies (Pan et al., 2024). This implies that, within Japan’s drug pricing mechanism, while the premium system addresses clinical value, the overall pricing framework remains only partially aligned with it. Clinical value is not sufficiently incorporated into the drug pricing formulation process.

This also suggests that the premium system has limited effectiveness and is unable to fully address the inherent limitations within the current pricing framework. This “price–value mismatch” partly reflects the inherent biases within Japan’s current drug pricing system. For drugs priced using the similar efficacy Comparative Method (I), the base price is determined by that of a comparator drug. When the comparator drug is priced low, even a drug granted a premium may be priced below its clinical value. For instance, trametinib 2 mg, approved for melanoma treatment, received a 45% clinical usefulness premium and an international price revision. However, due to the low price of its comparator drug, its final price was only 73.01% of the international average (MHLW, 2016).

For drugs priced using the Cost Calculation Method, the base price is derived from production, administrative, and other costs. Although this approach reflects objective cost inputs, it does not necessarily capture the drug’s full clinical value. Furthermore, to enhance pricing transparency, MHLW reformed the pricing system in 2022. The new policy stipulates that if the cost disclosure percentage of a drug is below 50%, it will lose its eligibility for a premium (MHLW, 2022).

While this reform improves transparency, it may also prevent drugs with high clinical value or innovation from receiving premiums simply due to insufficient cost disclosure. In our study, none of the eight drugs approved between 2022 and 2023 were affected by this regulation. However, Vyxeos, approved in 2024 for the treatment of acute myeloid leukemia, received a 45% clinical usefulness premium and a 10% market size premium but ultimately failed to receive any actual premium due to a cost disclosure percentage below 50%, resulting in a premium coefficient of 0 (MHLW, 2024).

These limitations largely restrict the alignment between drug prices and clinical value. This also suggests that, while ensuring the premium system reflects the clinical value of drugs, policymakers should place greater emphasis on the actual clinical values brought by the drugs in the final pricing process, and minimize the influence of non-clinical factors in pricing decisions.

Moreover, drug prices often fail to adequately reflect clinical value, a phenomenon not unique to Japan. A similar pattern has been observed in China. Although price negotiations have successfully reduced the costs of anticancer drugs, studies have found no significant correlation between price and clinical value before or after negotiations (Bao et al., 2023). In the United States, Michaeli et al. analyzed 145 anticancer drugs and found only a weak correlation between drug prices and clinical endpoints such as OS and PFS (Michaeli and Michaeli, 2023). Even in countries where cost-effectiveness evaluation has been introduced at the pricing stage, such as Italy, the mismatch between drug price and OS gain persists.

We also analyzed the differences in price revision magnitude and its relationship with clinical value. No statistically significant differences were observed between high clinical value and low/medium clinical value drugs in terms of either the magnitude of price revision or price reduction. This is primarily because drug price revision in Japan are chiefly influenced by pressures on healthcare expenditure and market sales performance, rather than the actual clinical value of drugs following their market launch. Should a drug’s annual sales significantly exceed projected figures, or should market competition intensify following the launch of generic equivalents, it may be subject to price revision regardless of its clinical value (MHLW, 2025a).

Further analysis of the price-adjusted medicines revealed a predominantly downward trend. This finding aligns with the current trajectory of pharmaceutical price revision in Japan (Pharmaceutical Technology, 2023). Moreover, no statistically significant difference was observed in the magnitude of price reductions between high-value medicines and those of medium to low value.

This price protection system is primarily based on the current price revision system for drugs in Japan. With universal health coverage and extensive drug reimbursement, healthcare expenditures remain under substantial pressure (Matsuda, 2019; Takayama and Narukawa, 2017). To alleviate the financial pressure and ensure the sustainability of the health insurance system, Japan adjusts the prices of drugs according to the survey of drug prices by the actual market transaction prices (MHLW, 2018), usually once every 2 years, and most of them are based on price reductions.

Although Japan permits medicines that receive innovative premiums to maintain their initial prices for a defined period, they are exempt from price reductions imposed through market mechanisms. This policy provides temporary price protection for drugs with higher clinical value and greater premium proportions, thereby moderating the speed of price declines.

However, this protection is conditional; once a medicine loses its eligibility for price protection. At that point, the drug undergoes a three-stage price revision: (1) retroactive correction to implement deferred price reductions; (2) alignment with current market prices to reflect actual supply-demand conditions; (3) removal of the premium initially granted for innovation.

This dynamic mechanism balances incentives and regulation across a drug’s life cycle—supporting innovation during early market entry and gradually guiding prices toward later-stage equilibrium. This approach helps manage healthcare spending while maintaining pricing rationality.

In recent years, in order to achieve the policy objective of controlling healthcare expenditures, Japan has continuously reduced the prices of medicines covered by NHI, changed the frequency of drug price adjustments from once every 2 years to once a year, and actively increased the proportion of generic medicines in the reimbursement list (Kinoshita and Kishimoto, 2024; Kosaka et al., 2023). Whilst these measures have alleviated pressure on the insurance fund in the short term, they have also given rise to a series of potential issues, including drug shortages and delays in the availability of essential medicines (BCCJ, 2025). Consequently, policymakers should reconsider establishing a relative protection mechanism for medicines of high clinical value during price revision. This would help prevent frequent price reductions from undermining pharmaceutical companies’ motivation for research and development, as well as compromising patients’ access to essential medicines.

A potential limitation of this study is that absolute improvements in PFS, OS, or ORR were used to assess clinical benefit, consistent with the ESMO-MCBS methodology. However, identical absolute gains may represent different clinical value depending on baseline prognosis. Future studies may consider incorporating relative percentage changes or standardized HR-based measures to improve comparability across indications.

This study has several limitations. Firstly, all clinical value assessments were based on pivotal clinical trials, which may not fully capture real-world effectiveness despite their recognized importance in pricing and revision decisions. Secondly, the pricing data reflects Japan’s standardized national prices and does not account for the potential impact of inflationary factors at the time of initial pricing. Thirdly, the findings have limited generalizability to specific indications or other indications not included in the analysis. Fourthly, this study did not conduct stratified analyses for tumour types with differing prognostic characteristics. We also recommend that future research undertake disease subgroup analyses based on this foundation to further validate the robustness of our findings. Finally, this study has not yet systematically considered the potential impact of disease severity and rarity on drug value and pricing. Future research will incorporate additional relevant variables into the existing analytical framework to broaden the scope of analysis and further test the robustness and scientific validity of the findings.

## 5 Conclusion

Overall, at the initial pricing stage, anticancer drugs that receive a premium typically demonstrate higher clinical value, and the magnitude of the premium can partially reflect this value. However, the absence of a significant correlation between treatment costs and clinical values indicates a persistent “price–value mismatch,” suggesting that the current pricing system’s ability to capture clinical value accurately remains limited. Additionally, certain high-clinical-value drugs receive limited protection during price revision. Our findings suggest that Japan’s current pricing and revision framework has shown some effectiveness in identifying and safeguarding high-clinical-value drugs. Nonetheless, there remains room for improvement, and policymakers are encouraged to review and refine pricing policies to align drug prices with their clinical value better.

## Data Availability

Publicly available datasets were analyzed in this study. This data can be found here: https://www.pmda.go.jp/review-services/drug-reviews/review-information/p-drugs/0010.html.
