# Supplementary material for: Pricing, price revision, and clinical value of anticancer drugs in Japan: a retrospective observational study
Source: Front Pharmacol. 2025 Nov 18;16:1641775. doi: 10.3389/fphar.2025.1641775 (PMC12668999; doi:10.3389/fphar.2025.1641775)
Supplement: Supplementary file 1 [file DataSheet1.pdf]

ESMO-MCBS scoring and mapping methodology

We applied the validated ESMO-MCBS to evaluate the indications of drugs at the time of their first approval. For solid tumors, we directly adopted the most recent ESMO-MCBS scores, as published on the ESMO website. For hematological malignancies, we used the versions published by the ESMO working group. Since the ESMO website is updated regularly, when the latest ESMO-MCBS scores for certain indications were not yet available, we conducted our own assessments based on the most up-to-date clinical trial evidence, using the official ESMO-MCBS evaluation forms and online tutorials. According to the official ESMO-MCBS framework, higher scores indicate greater clinical benefit and are interpreted as higher clinical value. Accordingly, in this study, scores of 4–5 (or grades A/B in the curative setting) were defined as high clinical value, while scores of 1–3 (or grade C in the curative setting) were defined as low-to-moderate clinical value.

Among the 94 indications we evaluated, 56 had ESMO-MCBS scores directly available from the ESMO website, including 49 solid tumors and 7 hematological malignancies. For the remaining 38 indications, the scores were assessed by the authors after studying the official ESMO online tutorials and applying the ESMO-MCBS evaluation forms, including 9 solid tumors and 29 hematological malignancies.

To avoid potential sample bias and ensure the integrity of cross-indication comparisons, drugs with curative intent were not excluded from the analysis. Instead, we applied a harmonized mapping approach to align the scoring systems used for curative and non-curative treatment settings. During the mapping process, key factors such as primary clinical outcomes from pivotal trials, toxicity profiles, and adverse events were comprehensively considered to derive an integrated score on a unified scale, thereby enabling comparability of drugs across different therapeutic contexts.

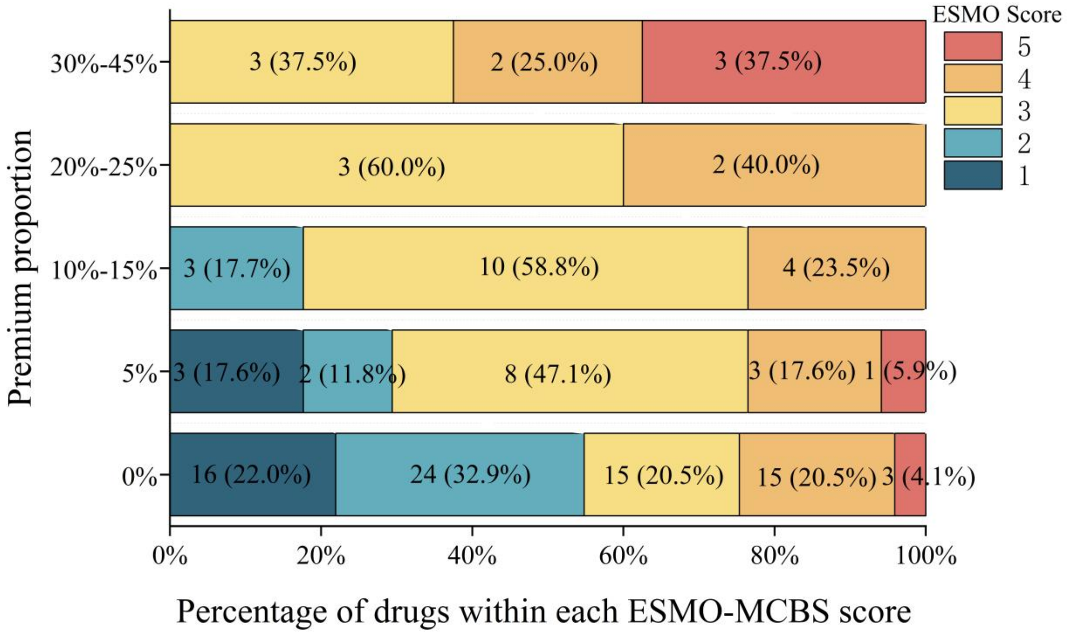

FigS1. Distribution of ESMO-MCBS scores for anticancer drugs with different premium proportions in Japan. The ESMO-MCBS scores were determined based on the latest scale and evaluation criteria published on the official ESMO website.

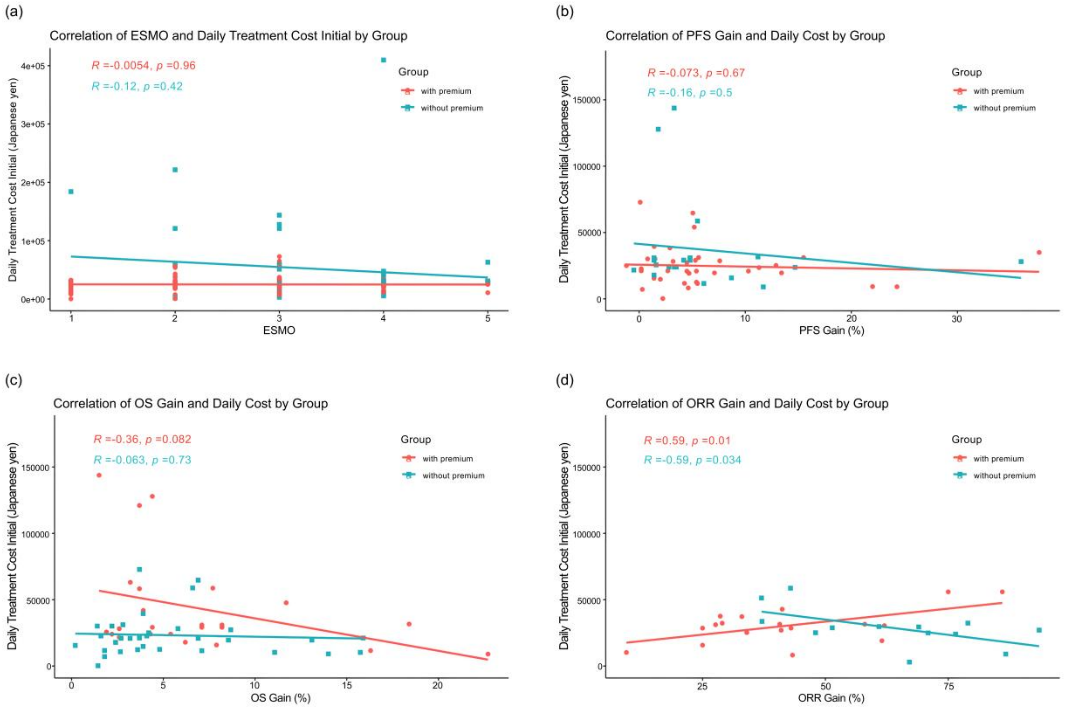

FigS2.Correlation between cancer therapy daily costs and percentage improvement for the premium and non-premium groups in (a) ESMO scores, (b) progression-free survival (PFS)

of randomized controlled trials, (c) overall survival (OS) of randomized controlled trials, and (d) objective response rate (ORR) of single-arm trials.

Table S1 Raw data on the basic characteristics and clinically relevant indicators of the study drug samples in pivotal clinical trials

[a] Drug: Refers to newly approved anticancer drugs for their first indication by PMDA between January 2013 and March 2023.

[b] PFS gain: Progression-Free Survival gain, referring to the absolute difference in median progression-free survival between the experimental group and the control group.

[c] OS gain: Overall Survival gain, referring to the absolute difference in median overall survival between the experimental group and the control group.

[d] ORR gain: Objective Response Rate gain, referring to the absolute difference in objective response rate (proportion of patients with complete response + partial response) between the experimental group and the control group.

[e] ESMO-MCBS: European Society for Medical Oncology - Magnitude of Clinical Benefit Scale.

Drug[a]	Approval year	Drug Indication	Pivotal trial ID	Clinical trail phase	Type of pivotal trial	PFS gain[b] （%）	OS gain[c] （%）	ORR gain[d] （%）	ESMO-MCBS[e]
Abemaciclib	2018	Breast cancer	NCT02246621	Phase III	Randomized	13.42	13.10	19.10	3
			NCT02107703	Phase III	Randomized	7.10	8.55	12.70	4
Abiraterone	2014	Prostate cancer	NCT00638690	Phase III	Randomized	2.00	3.90	NA	4
Acalabrutinib	2021	Lymphoma	NCT03198650	Phase I or II	Single-arm	7.00	NA	62.00	2
Afatinib	2014	NSCLC	NCT00949650	Phase III	Randomized	4.27	1.80	33.00	4
Aflibercept	2017	Colorectal cancer	NCT00561470	Phase III	Randomized	2.23	1.44	8.70	1
Alectinib	2014	NSCLC	JPRN-JapicCTI-101264	Phase I or II	Single-arm	NA	NA	93.50	3
Alemtuzumab	2014	Leukaemia	NCT00046683	Phase III	Randomized	2.90	NA	27.80%	2
Apalutamide	2019	Prostate cancer	NCT01946204	Phase III	Randomized	24.30	14.00	NA	4
Asciminib	2022	Leukaemia	NCT03106779	Phase III	Randomized	NA	NA	NA	3
Atezolizumab	2018	NSCLC	NCT02008227	Phase III	Randomized	NA	4.20	1	5
Avelumab	2017	Merkel Cell	NCT02155647	Phase I or II	Single-arm	2.70	12.60	33.00	3

		Carcinoma							
Bexarotene	2016	Lymphoma	JPRN-UMIN000006744	Phase I or II	Single-arm	NA	NA	61.50	2
Binimetinib	2019	Melanoma	NCT01909453	Phase III	Randomized	5.30	NA	12.00	5
Blinatumomab	2018	Leukaemia	NCT02412306	Phase I or II	Single-arm	13.10	14.80	NA	2
			NCT02013167	Phase III	Randomized	NA	3.70	NA	3
Bosutinib	2014	Leukaemia	NCT00261846	Phase I or II	Single-arm	NA	NA	NA	1
Brentuximab Vedotin	2014	Lymphoma	NCT00866047	Phase I or II	Single-arm	14.60	NA	86.00	3
			NCT00848926	Phase I or II	Single-arm	5.60	5.60	75.00	2
Brigatinib	2021	NSCLC	NCT03410108	Phase I or II	Single-arm	NA	NA	12.20	2
			NCT02737501	Phase III	Randomized	12.90	NA	34.00	4
Cabazitaxel	2014	Prostate cancer	NCT00417079	Phase III	Randomized	1.40	2.40	NA	2
Cabozantinib	2020	Renal cell carcinoma	NCT01835158	Phase I or II	Randomized	3.30	5.40	14.00	3
			NCT01865747	Phase III	Randomized	3.50	4.30	11.00	3
Capmatinib	2020	NSCLC	NCT02414139	Phase I or II	Single-arm	NA	NA	41.00	2
Carfilzomib	2016	Multiple Myeloma	NCT01080391	Phase I or II	Randomized	8.70	7.90	20.40	3
Cemiplimab	2022	Cervical cancer	NCT03257267	Phase III	Randomized	NA	3.20	10.80	5
Ceritinib	2016	NSCLC	NCT01283516	Phase I or II	Single-arm	7.00	NA	26.70	2
			NCT01685060	Phase I or II	Single-arm	5.80	15.60	40.70	1
Chidamide	2021	Lymphoma	NCT02955589	Phase I or II	Single-arm	7.60	34.40	NA	1
Clofarabine	2013	Leukaemia	NCT00317642	Phase III	Randomized	NA	0.20	NA	2
Dabrafenib	2016	Melanoma	NCT01584648	Phase III	Randomized	1.40	7.10	NA	5

			NCT01597908	Phase III	Randomized	4.80	8.20	15.00	3
			NCT01227889	Phase III	Randomized	4.20	4.40	15.70	4
Dacomitinib	2019	NSCLC	NCT01774721	Phase III	Randomized	5.50	7.10	3.30	3
Daratumumab	2017	Multiple Myeloma	NCT02076009	Phase III	Randomized	NA	15.74	NA	4
			NCT02136134	Phase III	Randomized	NA	11.07	19.70	4
Daratumumab Injection	2021	Multiple Myeloma	NCT03277105	Phase III	Randomized	NA	2.60	3.90	1
Darinaparsin	2022	Lymphoma	NCT02653976	Phase I or II	Single-arm	17.40	17.40	NA	1
Darolutamide	2020	Prostate cancer	NCT02200614	Phase III	Randomized	22.00	NA	NA	3
Denileukin diftitox	2021	Lymphoma	NCT02676778	Phase I or II	Single-arm	2.14	NA	41.20	2
Dinutuximab	2021	Neuroblastoma	NCT00026312	Phase III	Single-arm	NA	NA	NA	4
Durvalumab	2018	NSCLC	NCT02125461	Phase III	Randomized	11.20	18.40	12.80	4
Elotuzumab	2016	Multiple Myeloma	NCT01239797	Phase III	Randomized	4.50	8.68	13.00	2
Encorafinib	2019	Melanoma	NCT01909453	Phase III	Randomized	7.60	NA	23.00	4
Enfortumab vedotin	2021	Urothelial carcinoma	NCT03474107	Phase III	Randomized	NA	3.91	22.70	4
Entrectinib	2019	NSCLC	NCT02568267	Phase I or II	Single-arm	15.70	NA	67.10	3
Enzalutamide	2014	Prostate cancer	NCT00974311	Phase III	Randomized	5.40	4.80	NA	4
Forodesine	2017	Lymphoma	NCT01776411	Phase I or II	Single-arm	1.90	15.60	25.00	1
Gilteritinib	2018	Leukaemia	NCT02421939	Phase III	Randomized	NA	3.70	NA	3
Ibrutinib	2016	Lymphoma	NCT01578707	Phase III	Randomized	36.00	2.60	38.50	5
Inotuzumab ozogamicin	2018	Leukaemia	NCT01564784	Phase III	Randomized	3.30	1.50	NA	3

Ipilimumab	2015	Melanoma	NCT00094653	Phase III	Randomized	0.10	3.70	NA	3
			JPRN-JapicCTI-152869	Phase I or II	Single-arm	NA	NA	43.30	1
			NCT01844505	Phase III	Randomized	4.63	NA	13.00	3
Irinotecan	2020	Pancreatic cancer	NCT01494506	Phase III	Randomized	1.60	1.90	15.40	3
Isatuximab	2020	Multiple Myeloma	NCT02990338	Phase III	Randomized	5.06	6.90	30.00	3
Ixazomib	2017	Multiple Myeloma	NCT02181413	Phase III	Randomized	5.20	NA	NA	2
Larotrectinib	2021	Solid tumor	NCT02576431	Phase I or II	Single-arm	28.30	NA	79.00	3
Lenvatinib	2015	Thyroid carcinoma	NCT01321554	Phase III	Randomized	14.70	NA	NA	4
Leuprorelin	2015	Prostate cancer	NCT01546623	Phase III	Randomized	NA	NA	NA	2
Lorlatinib	2018	NSCLC	NCT03052608	Phase III	Randomized	NA	NA	18.00	4
Necitumumab	2019	NSCLC	NCT00981058	Phase III	Randomized	0.20	1.60	NA	1
	2019		JPRN-JapicCTI-132085	Phase I or II	Randomized	NA	4.10	2.40	3
Niraparib	2020	Epithelial Ovarian Cancer	NCT02354586	Phase I or II	Single-arm	3.50	17.20	NA	2
			NCT02655016	Phase III	Randomized	5.60	NA	NA	3
			NCT01847274	Phase III	Randomized	15.50	2.80	27.66	3
Nivolumab	2014	Melanoma	JPRN-jRCT2080221639	Phase I or II	Single-arm	5.63	18.00	28.60	2
Obinutuzumab	2018	Lymphoma	NCT03817853	Phase IV	Single-arm	NA	NA	86.70	3
			NCT01059630	Phase III	Randomized	11.70	22.70	NA	4
Ofatumuma	2013	Lymphoma	NCT00349349	Phase I or II	Single-arm	4.60	13.90	NA	2
Olaparib	2018	Breast cancer	NCT02000622	Phase III	Randomized	2.80	2.20	31.10	3

Osimertinib	2016	NSCLC	NCT02094261	Phase I or II	Single-arm	8.60	NA	70.90	3
Palbociclib	2017	Breast cancer	NCT01740427	Phase III	Randomized	10.30	2.70	NA	2
			NCT01942135	Phase III	Randomized	5.40	6.90	NA	4
Panobinostat	2015	Multiple Myeloma	NCT01023308	Phase III	Randomized	4.50	3.19	6.10	2
Pegfilgrastim	2014	Neutropenia	KRN125-008	Phase III	Randomized	NA	NA	NA	4
Pembrolizumab	2016	Melanoma	NCT01704287	Phase I or II	Randomized	0.20	3.70	3.10	3
			NCT01866319	Phase III	Randomized	2.70	15.90	21.80	4
Pemigatinib	2021	Biliary cancer	NCT02924376	Phase I or II	Single-arm	7.00	17.50	37.00	3
Pertuzumab	2013	Breast cancer	NCT00567190	Phase III	Randomized	6.10	16.30	10.90	4
Pimitespib	2022	Gastrointestinal Stromal Tumors	JPRN-JapicCTI-184094	Phase III	Randomized	1.40	6.20	NA	3
Polatuzumab vedotin	2021	Lymphoma	JPRN-JapicCTI-184048	Phase I or II	Single-arm	5.20	NA	42.90	2
			NCT02257567	Phase I or II	Randomized	5.50	7.70	NA	3
Pomalidomide	2015	Multiple Myeloma	NCT01311687	Phase III	Randomized	1.80	4.40	NA	3
Ponatinib	2016	Leukaemia	NCT01667133	Phase I or II	Single-arm	NA	NA	NA	1
			NCT01207440	Phase I or II	Single-arm	NA	6.90	NA	2
Pralatrexate	2017	Lymphoma	NCT02013362	Phase I or II	Single-arm	NA	NA	NA	1
			NCT00364923	Phase I or II	Single-arm	3.50	14.50	29.00	2
Quizartinib	2019	Leukaemia	NCT02039726	Phase III	Randomized	NA	6.60	NA	2
Ramucirumab	2015	Gastric cancer	NCT00917384	Phase III	Randomized	0.80	1.40	11.80	1
			NCT01170663	Phase III	Randomized	1.50	2.20	0.98	2

Regorafenib	2013	Colorectal cancer	NCT01103323	Phase III	Randomized	1.40	0.20	0.60	1
Romidepsin	2017	Lymphoma	NCT01456039	Phase I or II	Single-arm	5.60	NA	43.00	1
			NCT00426764	Phase I or II	Single-arm	NA	NA	25.00	2
Ropeginterferon alfa-2b-njft	2023	Polycythemia vera	NCT04182100	Phase I or II	Single-arm	NA	NA	NA	1
Ruxolitinib	2014	Myelofibrosis	NCT01392443	Phase I or II	Single-arm	NA	NA	NA	2
Selpercatinib	2021	NSCLC	NCT03157128	Phase I or II	Single-arm	NA	NA	69.00	3
Sotorasib	2022	NSCLC	NCT03600883	Phase I or II	Single-arm	6.80	NA	37.10	3
Streptozocin	2014	Gastroenteropancreatic neuroendocrine neoplasms	NPC-10-1	Phase I or II	Single-arm	NA	NA	9.50	1
Tazemetostat	2021	Lymphoma	NCT03456726	Phase I or II	Single-arm	NA	NA	76.50	3
Tepotinib	2020	NSCLC	NCT02864992	Phase I or II	Single-arm	11.20	19.60	51.40	3
Thiotepa	2019	Solid tumor	Japic CTI-163433	Phase I or II	Single-arm	NA	NA	NA	1
Tirabrutinib	2020	Lymphoma	JPRN-JapicCTI-173646	Phase I or II	Single-arm	NA	NA	NA	2
Trabectedin	2015	Soft tissue sarcoma	JapicCTI-121850	Phase I or II	Randomized	4.70	NA	NA	3
Trametinib	2016	Melanoma	NCT01584648	Phase III	Randomized	1.40	7.10	15.00	5
			NCT01597908	Phase III	Randomized	4.80	8.20	15.70	4
Trastuzumab deruxtecan	2020	Breast cancer	NCT03248492	Phase I or II	Single-arm	16.40	NA	60.90	3
Trastuzumab	2013	Breast cancer	NCT00829166	Phase III	Randomized	3.20	5.80	12.80	4

Emtansine									
Tremelimumab	2022	Hepatocellular Carcinoma	NCT03298451	Phase III	Randomized	NA	2.66	NA	5
Trifluridine	2014	Colorectal cancer	NCT01607957	Phase III	Randomized	0.30	1.80	NA	3
Valemetostat	2022	Lymphoma	NCT04102150	Phase I or II	Single-arm	NA	NA	48.00	1
Vandetanib	2015	Medullary thyroid cancer	NCT00410761	Phase III	Randomized	11.30	NA	NA	2
Vemurafenib	2014	Melanoma	NCT01006980	Phase III	Randomized	1.42	3.90	43.00	4
Venetoclax	2019	Lymphoma	NCT02265731	Phase I or II	Single-arm	NA	NA	NA	3
			NCT02005471	Phase III	Randomized	37.70	NA	NA	4
177Lu-DOTATATE	2021	Gastroenteropancreatic neuroendocrine neoplasms	NCT01578239	Phase III	Randomized	NA	11.70	NA	4
223Ra-radium dichloride	2016	Prostate cancer	NCT01929655	Phase I or II	Single-arm	NA	12.50	NA	1
			NCT00699751	Phase III	Randomized	NA	3.60	NA	4

Notes: NSCLC, non-small cell lung cancer.

Table S2 Raw data on the price, treatment cost, price change, and related characteristics of the study drug samples

[a] Drug: Refers to newly approved anticancer drugs for their first indication by PMDA between January 2013 and March 2023.

[b] Initial price: The price at which the drug was first listed in the National Health Insurance Drug Price List, with data obtained from the New Drug Pricing Table published by MHLW.

[c] Latest price: The current price of the drug as of April 2025 in the Japanese National Health Insurance Drug Price List.

[d] Price revision magnitude: The percentage change in price, calculated by comparing the latest price with the initial price.

[e] Premium rate: The premium percentage granted when the drug was first listed in the National Health Insurance Drug Price List, with data obtained from the New

Drug Pricing Table published by MHLW.

[f] Patient size: The estimated target treatment population size at the time of initial pricing, as assessed by MHLW and reported in the New Drug Pricing Table.

**Drug**[a]	**Initial price**[b]	**Daily treatment cost initial**	**Latest price**[c]	**Daily treatment cost latest**	**Price revision magnitude**[d]**(%)**	**Premium rate**[e]**(%)**	**Patient size**[f]	**Approval type**
Abemaciclib	3258.70	19552.20	3049.70	18298.20	-6.41	0	6200	Regular approval
Abiraterone	3690.90	14763.60	3759.30	15037.20	1.85	0	7500	Priority Review
Acalabrutinib	15202.20	30404.40	12921.90	25843.80	-15.00	0	309	Regular approval
Afatinib	5840.70	11681.40	4470.50	8941.00	-23.46	0	6900	Regular approval
Aflibercept	78614.00	227.98	65650.00	190.39	-16.49	0	5900	Regular approval
Alectinib	901.70	27051.00	901.70	27051.00	0	10	1500	Regular approval
Alemtuzumab	89254.00	38379.22	90907.00	39090.01	1.85	0	30	Regular approval
Apalutamide	2281.90	9127.60	2036.00	8144.00	-10.78	0	9200	Regular approval
Asciminib	5564.50	22258.00	5564.50	22258.00	0	10	583	Regular approval
Atezolizumab	625567.00	25022.68	563917.00	22556.68	-9.86	0	6300	Regular approval
Avelumab	218955.00	37222.35	166397.00	28287.49	-24.00	0	14	Regular approval
Bexarotene	2797.20	19020.96	2846.70	19357.56	1.77	0	300	Regular approval
Binimetinib	4836.80	29020.80	4926.40	29558.40	1.85	0	91	Regular approval
Blinatumomab	281345.00	120978.35	285961.00	122963.23	1.64	15	191	Regular approval
Bosutinib	3791.00	22746.00	3861.20	23167.20	1.85	0	1700	Regular approval
Brentuximab Vedotin	465701.00	55884.12	388958.00	46674.96	-16.48	0	300	Regular approval
Brigatinib	4200.50	25203.00	4176.10	25056.60	-0.58	0	670	Regular approval

Cabazitaxel	593069.00	17792.07	470502.00	14115.06	-20.67	0	640	Priority Review
Cabozantinib	8007.60	24022.80	8007.60	24022.80	0	10	2800	Regular approval
Capmatinib	5055.50	26945.82	5055.50	26945.82	0	0	443	Regular approval
Carfilzomib	23982.00	15828.12	24426.00	16121.16	1.85	10	1400	Regular approval
Cemiplimab	450437.00	63061.18	450437.00	63061.18	0	5	800	Regular approval
Ceritinib	6297.00	31485.00	6413.60	32068.00	1.85	0	530	Regular approval
Chidamide	20030.50	11417.39	20028.40	11416.19	-0.01	0	305	Regular approval
Clofarabine	140248.00	221591.84	146926.00	232143.08	4.76	10	58	Regular approval
Dabrafenib	4860.60	29163.60	4950.60	29703.60	1.85	45	80	Regular approval
Dacomitinib	3850.60	11551.80	3192.40	9577.20	-17.09	0	3400	Priority Review
Daratumumab	51312.00	10262.40	52262.00	10452.40	1.85	0	2000	Regular approval
Daratumumab Injection	434209.00	21710.45	445064.00	22253.20	2.50	5	6900	Regular approval
Darinaparsin	31692.00	28522.80	31692.00	28522.80	0	0	361	Regular approval
Darolutamide	2311.00	9244.00	2053.90	8215.60	-11.13	0	5400	Regular approval
Denileukin diftitox	85610.00	42805.00	85601.00	42800.50	-0.01	0	341	Regular approval
Dinutuximab	1365888.00	409766.40	1365888.00	409766.40	0	15	68	Regular approval
Durvalumab	112938.00	31622.64	67871.00	19003.88	-39.90	10	4200	Regular approval
Elotuzumab	160696.00	27318.32	162608.00	27643.36	1.19	0	2700	Regular approval
Encorafinib	3180.70	28626.30	3239.60	29156.40	1.85	0	91	Regular approval
Enfortumab vedotin	99609.00	41835.78	91444.00	38406.48	-8.20	10	3100	Priority Review
Entrectinib	5214.20	2972.09	5310.80	3027.16	1.85	20	142	SAKIGAKE

								Designation
Enzalutamide	3138.80	12555.20	2116.00	8464.00	-32.59	0	3100	Priority Review
Forodesine	2617.60	15705.60	2666.10	15996.60	1.85	0	335	Regular approval
Gilteritinib	19409.10	58227.30	19752.30	59256.90	1.77	15	506	SAKIGAKE Designation
Ibrutinib	9367.00	28101.00	8848.10	26544.30	-5.54	35	470	Regular approval
Inotuzumab ozogamicin	1307092.00	143780.12	1331297.00	146442.67	1.85	20	263	Regular approval
Ipilimumab	485342.00	72801.30	419578.00	62936.70	-13.55	0	220	Regular approval
Irinotecan	128131.00	25626.20	114410.00	22882.00	-10.71	10	1900	Regular approval
Isatuximab	64699.00	64699.00	64699.00	64699.00	0	0	769	Regular approval
Ixazomib	96519.00	54050.64	98306.40	55051.58	1.85	0	2200	Regular approval
Larotrectinib	4042.50	32340.00	4042.50	32340.00	0	5	117	Regular approval
Lenvatinib	3956.40	23738.40	3421.70	20530.20	-13.51	20	400	Regular approval
Leuprorelin	105039.00	624.98	66947.00	398.33	-36.26	0	140000	Regular approval
Lorlatinib	7216.40	28865.60	7350.00	29400.00	1.85	5	1200	Conditional Approval
Necitumumab	238706.00	22677.07	228328.00	21691.16	-4.35	0	724	Regular approval
Niraparib	10370.20	31110.60	9316.80	27950.40	-10.16	0	2300	Regular approval
Nivolumab	150200.00	37550.00	27130.00	6782.50	-81.94	0	470	Regular approval
Obinutuzumab	450457.00	9009.14	458799.00	9175.98	1.85	20	5500	Regular approval
Ofatumuma	27590.00	1931.30	28904.00	2023.28	4.76	10	140	Regular approval
Olaparib	3996.00	23976.00	3225.10	19350.60	-19.29	5	1200	Regular approval

Osimertinib	12482.50	24965.00	9670.00	19340.00	-22.53	5	2400	Priority Review
Palbociclib	5576.40	20911.50	5076.80	19038.00	-8.96	0	5600	Regular approval
Panobinostat	36583.90	20852.82	37261.40	21239.00	1.85	0	1300	Regular approval
Pegfilgrastim	106660.00	5333.00	82672.00	4133.60	-22.49	5	57000	Regular approval
Pembrolizumab	84488.00	21122.00	63077.00	15769.25	-25.34	0	7300	Regular approval
Pemigatinib	25631.20	51262.40	25631.20	51262.40	0	45	58	Regular approval
Pertuzumab	231866.00	11593.30	206472.00	10323.60	-10.95	5	2500	Regular approval
Pimitespib	6265.00	17917.90	6265.00	17917.90	0	5	82	Regular approval
Polatuzumab vedotin	1364330.00	58666.19	1159681.00	49866.28	-15。00	5	2400	Regular approval
Pomalidomide	42624.80	127874.40	36902.00	110706.00	-13.43	5	1500	Regular approval
Ponatinib	6318.30	18954.90	6428.40	19285.20	1.74	0	780	Regular approval
Pralatrexate	89632.00	32267.52	91292.00	32865.12	1.85	0	407	Regular approval
Quizartinib	19694.90	58887.75	20059.60	59978.20	1.85	0	211	Regular approval
Ramucirumab	75265.00	30106.00	76659.00	30663.60	1.85	0	15000	Regular approval
Regorafenib	5424.30	16272.90	5682.60	17047.80	4.76	0	4800	Priority Review
Romidepsin	109753.00	28535.78	111785.00	29064.10	1.85	0	466	Regular approval
Ropeginterferon alfa-2b-njft	297259.00	8917.77	272587.00	8177.61	-8.30	0	1700	Regular approval
Ruxolitinib	3706.80	7413.60	4074.20	8148.40	9.91	0	1600	Regular approval
Selpercatinib	3680.00	29440.00	4066.20	32529.60	10.49	15	748	Regular approval
Sotorasib	4204.30	33634.40	4204.30	33634.40	0	45	485	Regular approval
Streptozocin	42531.00	10207.44	43310.00	10394.40	1.83	0	130	Regular approval

Tazemetostat	3004.60	24036.80	3004.00	24032.00	-0.02	5	286	Regular approval
Tepotinib	14399.00	28798.00	14399.00	28798.00	0	15	523	SAKIGAKE Designation
Thiotepa	189816.00	184121.52	193331.00	187531.07	1.85	5	33	Accelerated Review
Tirabrutinib	5067.40	30404.40	4307.30	25843.80	-15.00	0	119	Regular approval
Trabectedin	49307.00	19229.73	49368.00	19253.52	0.12	0	420	Regular approval
Trametinib	7731.70	30926.80	7874.90	31499.60	1.85	45	70	Regular approval
Trastuzumab deruxtecan	165074.00	29713.32	160543.00	28897.74	-2.74	5	1300	Conditional Approval
Trastuzumab Emtansine	235108.00	28212.96	235820.00	28298.40	0.30	0	2600	Priority Review
Tremelimumab	214801.00	10740.05	213831.00	10691.55	-0.45	0	3100	Regular approval
Trifluridine	2489.60	7120.26	2511.00	7181.46	0.86	0	8200	Regular approval
Valemetostat	6267.70	25070.80	6267.70	25070.80	0	5	101	Regular approval
Vandetanib	7836.40	23509.20	7902.20	23706.60	0.84	0	53	Regular approval
Vemurafenib	4935.50	39484.00	5026.90	40215.20	1.85	0	120	Regular approval
Venetoclax	874.60	34984.00	872.80	34912.00	-0.21	0	123	Regular approval
177Lu-DOTATATE	2648153.00	47666.75	2647734.00	47659.21	-0.02	10	205	Regular approval
223Ra-radium dichloride	684930.00	12328.74	697614.00	12557.05	1.85	0	3000	Regular approval
